# Genomic analysis of the slope of the reaction norm for body weight in Australian sheep

**DOI:** 10.1186/s12711-022-00734-6

**Published:** 2022-06-03

**Authors:** Dominic L. Waters, Sam A. Clark, Nasir Moghaddar, Julius H. van der Werf

**Affiliations:** School of Environmental & Rural Science, University of New England, Armidale, NSW 2351 Australia

## Abstract

**Background:**

Selection of livestock based on their robustness or sensitivity to environmental variation could help improve the efficiency of production systems, particularly in the light of climate change. Genetic variation in robustness arises from genotype-by-environment (G × E) interactions, with genotypes performing differently when animals are raised in contrasted environments. Understanding the nature of this genetic variation is essential to implement strategies to improve robustness. In this study, our aim was to explore the genetics of robustness in Australian sheep to different growth environments using linear reaction norm models (RNM), with post-weaning weight records of 22,513 lambs and 60 k single nucleotide polymorphisms (SNPs). The use of scale-corrected genomic estimated breeding values (GEBV) for the slope to account for scale-type G × E interactions was also investigated.

**Results:**

Additive genetic variance was observed for the slope of the RNM, with genetic correlations between low- and high-growth environments indicating substantial re-ranking of genotypes (0.44–0.49). The genetic variance increased from low- to high-growth environments. The heritability of post-weaning body weight ranged from 0.28 to 0.39. The genetic correlation between intercept and slope of the reaction norm for post-weaning body weight was low to moderate when based on the estimated (co)variance components but was much higher when based on back-solved SNP effects. An initial analysis suggested that a region on chromosome 11 affected both the intercept and the slope, but when the GEBV for the slope were conditioned on the GEBV for the intercept to remove the effect of scale-type G × E interactions on SNP effects for robustness, a single genomic region on chromosome 7 was found to be associated with robustness. This region included genes previously associated with growth traits and disease susceptibility in livestock.

**Conclusions:**

This study shows a significant genetic variation in the slope of RNM that could be used for selecting for increased robustness of sheep. Both scale-type and rank-type G × E interactions contributed to variation in the slope. The correction for scale effects of GEBV for the slope should be considered when analysing robustness using RNM. Overall, robustness appears to be a highly polygenic trait.

**Supplementary Information:**

The online version contains supplementary material available at 10.1186/s12711-022-00734-6.

## Background

Genotype-by-environment (G × E) interactions occur when the expression of the genes carried by an individual changes depending on the environment in which it is raised. This means that the best animal for one environment might not necessarily be the best one in another environment. One approach to this problem is to design breeding programs for specific geographical environments [1]. However, within the same geographical location, environments often vary considerably from year to year, such as during periods of drought [2]. An alternative is to select animals that rank highly across wide-ranging environmental conditions. These animals are said to be robust, as their breeding value is less sensitive to the environment.

The robustness of an animal cannot be directly measured, since it is typically raised in a single location at a given time point. However, robustness can be inferred for a genotype using a reaction norm model (RNM) that includes genetic relationships between relatives that have been raised in different environments. In a RNM, the estimated breeding value (EBV) of each animal is modelled as a function of a continuous environmental descriptor using random regression. A linear function is typically used as it is easier to interpret than higher order polynomials [3]. This results in two EBV for each individual, i.e., an intercept, which captures the average performance across environments, and a slope, which captures the amount of change in EBV across the environmental descriptor. The EBV for the slope is directly treated as an EBV for robustness. Many previous studies have used RNM to model sensitivity to environmental changes [3, 4]. For example, Hollema et al. [5] used a linear RNM to model variation in the sensitivity of Australian sheep to parasite burden using pedigree relationships. The average worm-burden of a contemporary group was used as the environmental descriptor, and a flatter slope was interpreted as a more robust genotype.

More recently, RNM have been extended to include genomic information in the form of single nucleotide polymorphisms (SNPs) [6]. The use of genomic data increases the power for estimating robustness, since it captures the genetic linkage between distantly-related genotypes in different environments [7]. Compared to regular genomic best linear unbiased prediction (GBLUP) models that ignore G × E interactions, RNM models have shown increased predictive ability [8, 9]. When a reference population is well-distributed across environments, selection based on genomic estimated breeding values (GEBV) greatly improves the response to selection for robustness [10], as young sires can be selected earlier without the need for large progeny tests across multiple environments. In addition, genomic RNM can be used for genome-wide association (GWA) studies by back-solving GEBV to SNP effects. This can shed light on the molecular basis underlying robustness and improve the accuracy of genomic predictions by using models which incorporate the molecular information as priors [11].

The correlation between the genetic variance in the intercept and that in the slope has often been limited to a discussion as a correlation between overall performance and sensitivity [12]. Beyond this, a correlation implies that (1) scale changes in genetic variance occur across environments, and (2) information from the intercept is used to estimate the EBV for the slope (and vice versa). Both of these observations could influence the EBV for the slope but are unrelated to the re-ranking of genotypes. Therefore, it might be important to ensure a level of independence between the EBV for the slope and the EBV for the intercept in the analysis of robustness.

The objectives of this study were to: (1) estimate the genetic variation of robustness in Australian sheep between different growth environments using linear reaction norms models fitted by random regression; and (2) compare the use of direct and scale-corrected GEBV for the slope in a genome-wide association study, using post-weaning weight (PWWT) as the response variable.

## Methods

### Data structure

In this study, data from 34,584 sheep recorded in the Australian Sheep CRC Information Nucleus Flock (INF) and the Meat and Livestock Australia Resource Flock (RF) were used. The INF animals were located at eight sites across southern Australia between 2007 and 2011. Each year, approximately 100 sires from the two main sites (Armidale NSW and Katanning WA) were mated via artificial insemination, each one with 900 ewes. For the six remaining sites, 450 ewes were mated annually, with at least 50% of the Kirby/Katanning sires. Then from 2012 onwards, the program was changed: it used the RF and only the Armidale and Katanning sites were kept under a similar mating structure, but with about 150 sires mated at both sites each year. The management practices at each site reflected the typical practices used for raising lambs on pasture in each of the respective geographical areas. Supplemental feed was occasionally provided in years with periods of drought to help maintain the body weight of animals.

The population consisted of multiple breeds. Merino, Maternal (such as Border Leicester) or Terminal (such as Poll Dorset) sires were mated to either Merino or first cross Maternal-Merino dams. Only the Merino breed had pure-bred representatives, while the remaining breeds were represented as crosses. Overall, 39 different breed groups were available, referred to as genetic groups hereafter. A summary of the breed composition of the data is in Additional file 1: Table S1. For more information on the INF and RF flocks, see [13].

Phenotypic records for post-weaning growth rate (PWGR) and post-weaning weight (PWWT) were used. First, PWGR was used to estimate the environmental descriptor used in the RNM. The PWGR of each lamb was calculated as the difference between weaning and post-weaning weights, recorded at approximately 96 (64–120) and 238 (120–329) days of age, respectively, which was divided by the number of days between measurements and expressed in g/day. PWWT was then used as the dependent variable for the RNM analysis. The distribution of PWGR and PWWT as well as the other descriptive statistics after filtering are available in Additional file 2: Table S2.

### Preparation of the reaction norm data

Since explicit environmental information was not available for the each of the sites, the best linear unbiased estimation (BLUE) of the contemporary group effect for PWGR was used to estimate the environmental descriptor. Contemporary groups (CG) were formed as a cohort of site $\times$ birth year $\times$ management group. The management group consisted of animals which were subjected to the same management decisions within each site $\times$ year, i.e., they were raised in the same paddocks and phenotyped at the same time. PWGR was chosen as the environmental descriptor because rate of growth has an intuitive relationship with environmental quality, i.e., CG that grow more quickly after weaning, are more likely to be in a better environment than groups that grow more slowly. When implemented in a reaction norm model, the slope of a genotype is interpreted as the change in GEBV per change in growth environment (g/day). Other studies have also demonstrated the value in using alternative traits for the environmental gradient (EG), rather than the trait used as the reaction norm response variable (i.e., PWWT) [14].

Lambs that had a growth period shorter than 40 days, a PWGR more than 4 standard deviations (SD) from the mean PWGR or that were born as quintuplets or reared as quadruplets were not included in the analysis. Only CG with a minimum of 15 animals were considered. After filtering, 33,773 individuals from 249 CG were available to estimate the CG effects. The model for obtaining the BLUE of CG effects was a regular pedigree-based animal model:

1 $$\mathbf{y}=\mathbf{X}\mathbf{b}+{\mathbf{Z}}_\mathbf{1}\mathbf{a}+\mathbf{Q}\mathbf{g}+\mathbf{e},$$

where $\mathbf{y}$ is the vector of PWGR records, $\mathbf{X}$ is an incidence matrix for the fixed effects $\mathbf{b}$, ${\mathbf{Z}}_\mathbf{1}$ is an incidence matrix relating the records to additive genetic effects ($\mathbf{a}$), $\mathbf{Q}$ is a matrix of the proportion of each animal’s genome originating from the 39 genetic groups [15], $\mathbf{g}$ is the vector of the random genetic group effects, and $\mathbf{e}$ is the vector of the residual effects. Fixed effects included sex, birth-type and rear-type interaction, age at post-weaning (linear and quadratic) and CG.

Only the CG effects for genotyped animals (22,634) were extracted. Each animal was assigned the BLUE of their CG as their environmental descriptor, which was then standardised to have a mean of 0 and variance of 1, forming the EG used in the RNM.

This subset of genotyped animals was re-filtered to ensure that the animals had a PWWT within 4 SD from the mean PWWT, originated from CG of at least 15 other animals, or had at least one half-sib. After filtering, 22,513 animals from 1582 sires and 11,576 dams across 206 CG were available for RNM analysis.

The CG effects ranged from − 2.15 SD (− 72.4 g/day) to 2.06 SD (69.1 g/day) respectively, with an overall range of 141.5 g/day. On average, sires had progeny in environments with PWWT differing by 59 g/day. A small number of the sires (6%) had progeny in only one environment, but these were still included since their genomic data can link them to other animals across environments. The distribution of records and mean PWWT along the EG is plotted in Additional file 3: Figure S1.

### Genotype data

Not all animals were genotyped due budget constraints. Genotyped animals were selected randomly from each sire to minimise selection bias. The imputed genomic data for each individual consisted of 60,400 SNPs. Originally, animals were genotyped with a 12 k, or one of two 15 k or one of two 50 k SNP panels. Approximately 93% of the animals were genotyped with one of the 50 k SNP panels. Within each panel, SNPs were removed if they were located on the X or Y chromosome, deviated greatly from the Hardy–Weinberg equilibrium (P < 10^–15^), had call rates lower than 90% and Gen-Cal scores lower than 0.6. When the correlation between the genotype sample of an animal and that of another animal was higher than 0.98, they were removed, as this indicated an error in matching ID. Following quality control, the Beagle 5.1 software [16] was used to impute all SNP genotypes to the combined set of 60,400 SNPs. The number of reference animals used to impute the SNP genotypes for each chip is in Additional file 4: Table S3. After imputation, the combined set was filtered to remove SNPs with a minor allele frequency lower than 0.01. Finally, 60,347 SNPs were available for analysis.

### Reaction norm analysis

Two RNM were used to estimate GEBV. The first RNM (RNM-HOM) assumed that the residual variance was homogenous along the EG, and is described by:

2 $$\mathbf{y}=\mathbf{X}\mathbf{b}+{\mathbf{Z}}_\mathbf{1}{\mathbf{a}}_\mathbf{1}+{\mathbf{Z}}_\mathbf{2}{\mathbf{a}}_\mathbf{2}+\mathbf{Q}\mathbf{g}+\mathbf{e},$$

where $\mathbf{y}$ is the vector of PWWT records, $\mathbf{X}$ is an incidence matrix linking records to the fixed effects $\mathbf{b}$, and ${\mathbf{Z}}_\mathbf{1}$ and ${\mathbf{Z}}_\mathbf{2}$ are incidence matrices linking records to the breeding values for the intercept $({\mathbf{a}}_\mathbf{1})$ and the slope $({\mathbf{a}}_\mathbf{2})$, $\mathbf{Q}$ is a matrix of the proportion of each animal’s genome originating from the 39 genetic groups, $\mathbf{g}$ is the vector of the random genetic group effects, and $\mathbf{e}$ is the vector of residual effects. Fixed effects included sex, birth type, rear type, age at post-weaning and CG. The $\mathbf{Q}$ matrix and CG were formed as previously described for the BLUE model.

The genetic variance of ${\mathbf{a}}_\mathbf{1}$ and ${\mathbf{a}}_\mathbf{2}$ was modelled according to $$\left[\begin{array}{c}{\mathbf{a}}_\mathbf{1}\\ {\mathbf{a}}_\mathbf{2}\end{array}\right]\sim N\left(\mathbf{0},\mathbf{G}\otimes \mathbf{K}\right)$$ where $$\mathbf{K}=\left[\begin{array}{c}{ \sigma }_{{a}_{1} }^{2}{ \sigma }_{{{a}_{2}a}_{1}} \\ { \sigma }_{{{a}_{1}a}_{2}} {\sigma }_{{a}_{2} }^{2}\end{array}\right]$$ and $\mathbf{G}$ is a relationship matrix based on genomic data, constructed following the first method described by VanRaden [17].

Genetic groups were modelled as for Eq. (1). The variance of the residual $\mathbf{e}$ was modelled according to $\left[\mathbf{e}\right]\sim N\left(\mathbf{0},\mathbf{I}{\sigma }_{e}^{2}\right)$, where ${\sigma }_{e}^{2}$ is the residual variance which was assumed to be homogenous across environments.

The second RNM (RNM-HET) was the same as RNM-HOM, except that residual variance was modelled as a function of the EG, as described in [18]. An intercept (${\mathbf{e}}_\mathbf{1})$ and slope (${\mathbf{e}}_\mathbf{2})$ residual coefficient was used, such that $$\left[\begin{array}{c}{\mathbf{e}}_\mathbf{1}\\ {\mathbf{e}}_\mathbf{2}\end{array}\right]\sim N\left(\mathbf{0},\mathbf{I}\otimes \mathbf{E}\right)$$, where $$\mathbf{E}=\left[\begin{array}{cc}{ \sigma }_{{e}_{1} }^{2}& { \sigma }_{{{e}_{2}e}_{1}}\\ { \sigma }_{{{e}_{1}e}_{2}}& {\sigma }_{{e}_{2} }^{2}\end{array}\right]$$. The models were implemented using genomic residual maximum likelihood (GREML) with the MTG2 software [19].

The genetic (co)variance matrix for breeding values between environments along the EG were estimated as: $\widehat{\mathbf{V}}={\varvec{\Lambda}}\mathbf{K}{\varvec{\Lambda}}\mathrm{^{\prime}}$, where $\mathbf{K}$ is the additive genetic (co)variance matrix for the intercept and the slope, and ${\varvec{\Lambda}}$ is a 100 × 2 matrix containing a vector of 1s for the intercept and a vector of 100 standardised environmental values ranging from the minimum to the maximum value of the EG. The genetic correlation matrix between environments was computed from $\widehat{\mathbf{V}}$.

For RNM-HET, the residual variance for environment at level $i$ was calculated as the element $i,$ in the matrix $\widehat{\mathbf{R}}$*,* which was estimated as: $\widehat{\mathbf{R}}={\varvec{\Lambda}}\mathbf{E}{\varvec{\Lambda}}\mathbf{^{\prime}}$**,** where $\mathbf{E}$ is the (co)variance matrix for the residual intercept and slope regression coefficients, and ${\varvec{\Lambda}}$ is the same as above.

The heritability of PWWT in environment $i$ was estimated as: ${h}_{i}^{2}=\frac{{V}_{{a}_{i}}}{{V}_{{a}_{i}}+{V}_{{e}_{i}}}$, where ${V}_{{a}_{i}}$ is the additive genetic varaince estimated for environment $i$ and ${V}_{{e}_{i}}$ is the residual variance in environment $i$*.* Standard errors for the variance components and heritabilities were estimated using a Taylor series expansion [20].

To observe how parameters changed along the EG, the genetic variance, correlation and heritabilites from the two models were compared betweem three distinct environments: − 1.5 SD, 0 SD and 1.5 SD, refering to low-, average- and high-growth environments, respectively. Evaluation of changes in parameters was limited to this range of EG values, as estimates outside this range may be biased since they are based on relatively small amounts of data [21]. To further validate the reaction norm variance parameters, the data were split into three sections based on the EG: low (< − 1.13 SD), average (> 0.59 SD and < 0.59 SD) and high (> 1.13 SD), such that the mean EG of each section was − 1.55, 0.06 and 1.52 SD for low, average and high sections, respectively. In total, 3157, 9375 and 3384 animals were included in the low, average and high sections, respectively. A multi-trait model (MTM), which considered performance in the three sections as different traits but allowed them to be genetically correlated, was then fitted. This provided a robust estimate of the parameters for the three distinct environments, which could be used as a benchmark for the reaction norms, as done in [22].

### Genome-wide association (GWA) analysis

To obtain scale-corrected GEBV for the slope (${\mathbf{a}}_\mathbf{2}^{\mathbf{*}})$ from RNM-HOM and RNM-HET for GWA analysis, the following genetic regression was used:

3 $${\mathbf{a}}_\mathbf{2}^{\mathbf{*}}={\mathbf{a}}_\mathbf{2}-\frac{{\sigma }_{{a}_{1}{a}_{2}}}{{\sigma }_{{a}_{1}}^{2}}{\mathbf{a}}_\mathbf{1},$$

where ${\mathbf{a}}_\mathbf{1}$ and ${\mathbf{a}}_\mathbf{2}$ are the GEBV for the intercept and the slope, respectively, ${\sigma }_{{a}_{1}}^{2}$ is the variance in intercept and ${\sigma }_{{{a}_{1}a}_{2}}$ is the covariance between intercept and slope, estimated from the respective RNM models. The aim of this formula was to remove the variation in GEBV for the slope that originated from scale effects. To distinguish the different GEBV for the slope, the terms direct slope (${\mathbf{a}}_\mathbf{2}$) and scale-corrected slope (${\mathbf{a}}_\mathbf{2}^{\mathbf{*}}$) are used.

The GWA analyses were performed by separately back-solving the GEBV for the intercept (${\mathbf{a}}_\mathbf{1}$), the direct slope (${\mathbf{a}}_\mathbf{2}$) and scale-corrected slope (${\mathbf{a}}_\mathbf{2}^{\mathbf{*}}$) to SNP effects for each model, following [23]:

4 $$\widehat{\mathbf{u}}=\frac{{\mathbf{W}\mathbf{G}}^{\mathbf{-1}}\mathbf{a}}{d},$$

where $\widehat{\mathbf{u}}$ is the vector of SNP effects, $d$ is a scalar calculated as $2\sum_{i=1}^{60347}p(1-p)$, where $p$ is the allele frequency for SNP $i$, $\mathbf{W}$ is the SNP matrix corrected for allele frequency differences ($\mathbf{W}=\mathbf{M}-2*(p-0.5)$, $\mathbf{M}$ is the SNP matrix coded such that 0 represents the heterozygous genotype, and − 1 and 1 represents the homozygous genotypes), ${\mathbf{G}}^\mathbf{{-1}}$ is the inverse of the genomic relationship matrix [17], and $\mathbf{a}$ is the vector of GEBV (either ${\mathbf{a}}_\mathbf{1}$, ${\mathbf{a}}_\mathbf{2}$, or ${\mathbf{a}}_\mathbf{2}^{\mathbf{*}}$).

An approximate *p*-value of each SNP effect was estimated based on a t-distribution, calculated as the probability of the 95th percentile of the GEBV distribution [24]. A Bonferroni correction was used to avoid false positives. A SNP was considered significant when its *p*-value was lower than 0.05/60,347, giving a threshold of − log_10_(*p*) > 6.08. Genes in the genomic regions that were significantly associated with the scale-corrected slope were detected based on the *Ovis aries* v3.1 assembly using the NCBI genome data viewer [25].

## Results

Additive genetic variation was observed for the slope, indicating the presence of G × E interactions and genetic variation in robustness for PWWT (Table 1). The genetic correlation between the intercept and the slope, obtained from the additive genetic (co)variance components, was much lower in RNM-HET (0.18) than in RNM-HOM (0.52). Overall, RNM-HET provided a better fit based on the log-likelihood, which indicated that the residual variance was heterogenous along the EG.

**Table 1:** Variance of the additive genetic intercept **(**${{\varvec{\sigma}}}_{{\varvec{a}}\mathbf{1}\boldsymbol{ }}^\mathbf{2}$**)** and slope **(**${{\varvec{\sigma}}}_{{\varvec{a}}\mathbf{2}\boldsymbol{ }}^\mathbf{2}$**)**, the residual intercept **(**${{\varvec{\sigma}}}_{{\varvec{e}}\mathbf{1}\boldsymbol{ }}^\mathbf{2}$**)**, slope **(**${{\varvec{\sigma}}}_{{\varvec{e}}\mathbf{2}\boldsymbol{ }}^\mathbf{2}$**)** and covariance **(**${{\varvec{\sigma}}}_{{\varvec{e}}\mathbf{1}\boldsymbol{ }}{{\varvec{\sigma}}}_{{\varvec{e}}\mathbf{2}}$**)**, as well as genetic group (g) variance

Model^a^	${{\varvec{\sigma}}}_{{\varvec{a}}\mathbf{1}\boldsymbol{ }}^\mathbf{2}$	${{\varvec{\sigma}}}_{{\varvec{a}}\mathbf{2}\boldsymbol{ }}^\mathbf{2}$	${{\varvec{r}}}_{{\varvec{a}}\mathbf{1}{\varvec{a}}\mathbf{2}}$	${{\varvec{\sigma}}}_{{\varvec{e}}\mathbf{1}\boldsymbol{ }}^\mathbf{2}$	${{\varvec{\sigma}}}_{{\varvec{e}}\mathbf{2}\boldsymbol{ }}^\mathbf{2}$	${{\varvec{\sigma}}}_{{\varvec{e}}\mathbf{1}{\varvec{e}}\mathbf{2}}$	g	LKH
RNM-HOM	7.40 (0.31)	1.28 (0.16)	0.52 (0.04)	17.52 (0.26)	–	–	11.45 (3.85)	− 46,938.28
RNM-HET	6.98 (0.31)	1.19 (0.17)	0.18 (0.06)	18.17 (0.31)	− 0.32 (0.23)	1.55 (0.14)	10.88 (3.69)	− 46,870.32^b^

The correlation between additive genetic intercept and slope (${r}_{{\varvec{a}}\mathbf{1}{\varvec{a}}\mathbf{2}}$) and the log-likelihood (LKH) are also reported

^a^RNM-HOM: linear reaction norm model with homogenous residual variance; RNM-HET: linear reaction norm model with heterogenous residual variance

^b^Based on a log-likelihood ratio test using a chi-square distribution, RNM-HET provided a better fit (p = 1.7 × 10^15^)

**Fig. 1:**
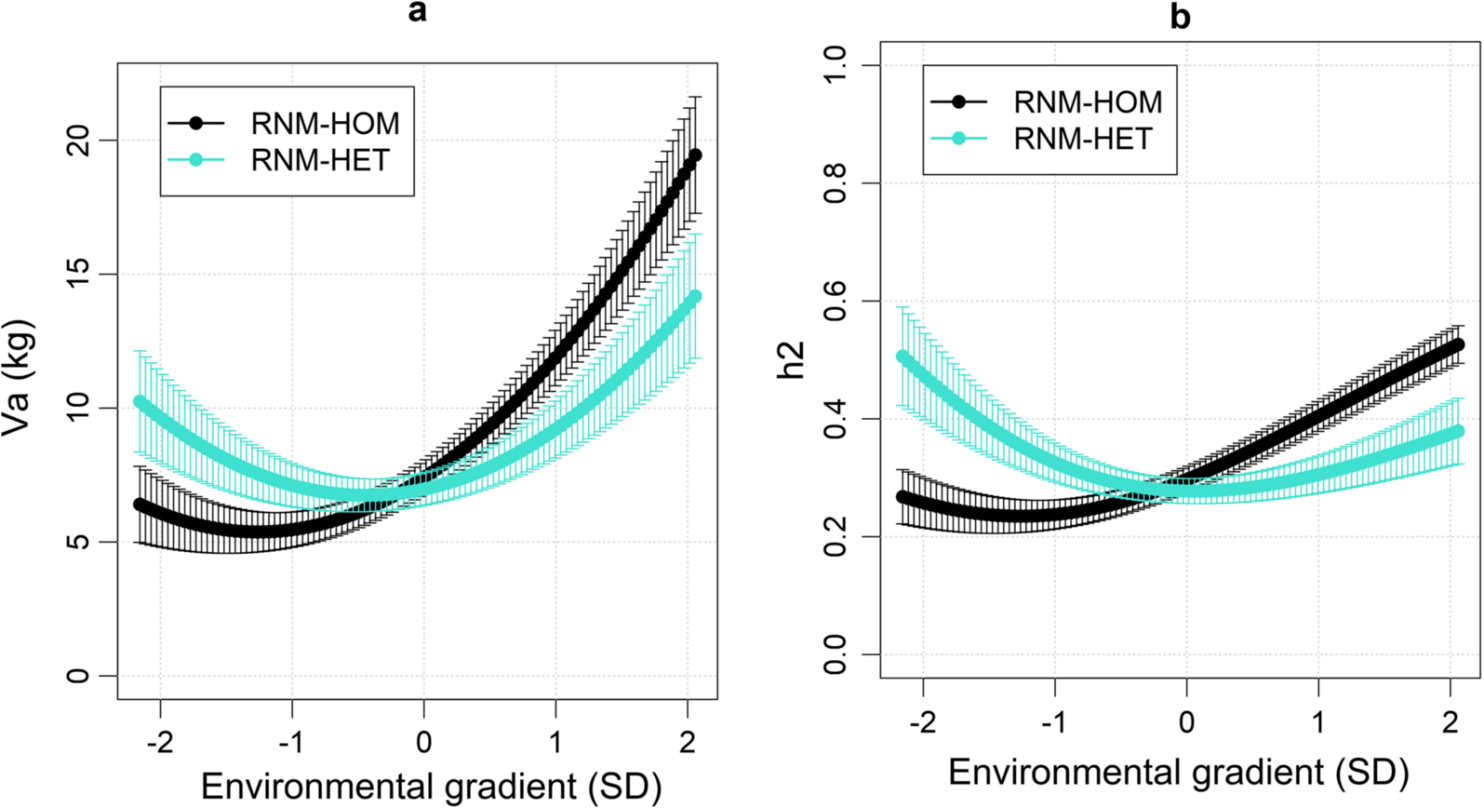
Genetic variance and heritability along the environmental gradient. Estimates of **a** genetic variance (${\mathrm{V}}_{\mathrm{a}}$) and **b** heritability (${h}^{2}$) with 95% confidence intervals along the environmental gradient. RNM-HOM: linear reaction norm model with homogenous residual variance, RNM-HET: linear reaction norm model with heterogenous residual variance

The genetic variance increased by 177% from low- to high-growth environments in RNM-HOM (Fig. 1a), but increased only by 39% across the same environments in RNM-HET, although the greatest increase in RNM-HET was between average- and high-growth environments (61%). This still showed substantially lower scale-type G × E interactions and indicated considerable bias in the genetic parameters of RNM-HOM.

The estimates of heritability in RNM-HOM followed the same pattern as the genetic variance, increasing by 92% from low- to high-growth environments (Fig. 1b). The heritability in RNM-HET was higher in low-growth environments (0.39) than in average- and high-growth environments (0.28–0.33). The residual variance increased by 74% from low- to high-growth environments in RNM-HET (see Additional file 5: Figure S2).

**Fig. 2:**
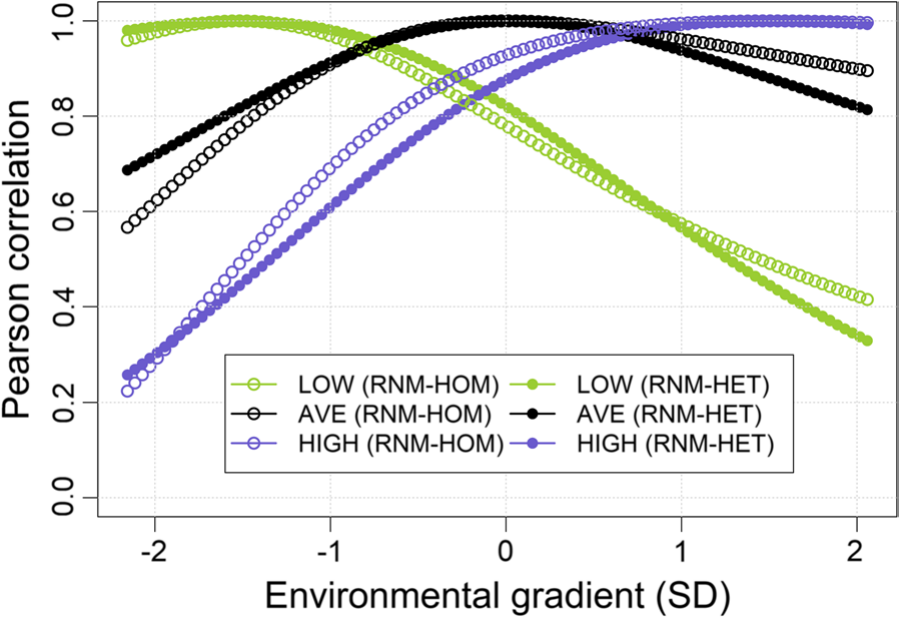
Genetic correlations between low-, average- and high-growth environments. Estimates of genetic correlations between low- (− 1.5 SD, green), average- (0 SD, black), and high- (1.5 SD, purple) growth environments from the two models. RNM-HOM: linear reaction norm model with homogenous residual variance, RNM-HET: linear reaction norm model with heterogenous residual variance

Genetic correlations between low- and high-growth environments were 0.49 and 0.44 in RNM-HOM and RNM-HET, respectively (Fig. 2), which indicated a high degree of rank-type G × E interactions along the EG and variation in robustness. Overall, both models produced similar estimates of rank-type G × E interactions.

The parameter estimates of RNM-HET were largely consistent with those of the MTM (see Additional file 6: Table S4). However, the genetic correlations obtained with the MTM were slightly lower between similar environments (Low vs Average, Average vs High) and higher between the less similar environments (Low vs High) than with RNM-HET. The parameter estimates of RNM-HOM were substantially different from those of the MTM, which suggests that RNM-HOM was biased.

## GWA analysis and scale correction

**Fig. 3:**
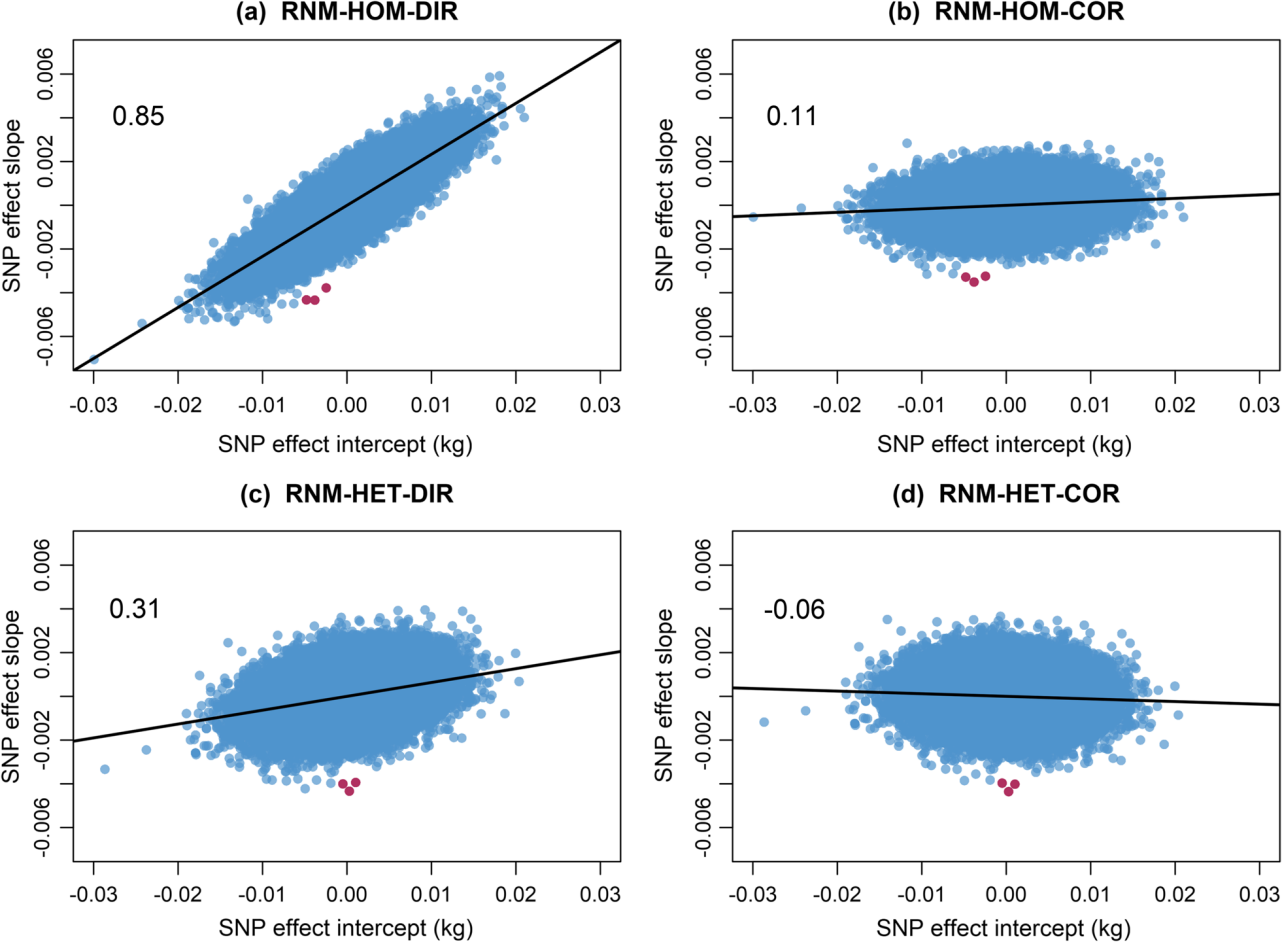
Scatterplots between direct and scale-corrected SNP effects for the slope against the intercept. Scatterplots and correlations between SNP effects for the direct slope (**a** and **c**) and scale-corrected slope (**b** and **d**) against the intercept. Significant SNPs on chromosome 7 are highlighted in red. RNM-HOM-DIR: linear reaction norm model with homogenous residual and direct breeding value for slope; RNM-HOM-COR: linear reaction norm model with homogenous residual and corrected breeding value for slope; RNM-HET-DIR: linear reaction norm model with heterogenous residual and direct breeding value for slope; RNM-HET-COR: linear reaction norm model with heterogenous residual and corrected breeding value for slope

The correlation between back-solved SNP effects for the intercept and the direct slope in RNM-HOM was high (0.85), indicating a large amount of scale-type G × E interactions (Fig. 3a). The correlation was lower in RNM-HET (0.31), but still suggested scale effects (Fig. 3c). These correlations were considerably higher than the genetic correlations based on the estimated (co)variance components for the intercept and the slope in both models in Table 1 (RNM-HOM 0.85 vs 0.52, RNM-HET 0.31 vs 0.18).

To estimate the scale-corrected SNP effects following Eq. (3), the correction formulas for the scale-corrected GEBV of RNM-HOM and RNM-HET were: ${\mathbf{a}}_\mathbf{2}^{\mathbf{*}}={\mathbf{a}}_\mathbf{2}-0.21{\mathbf{a}}_\mathbf{1}$ and ${\mathbf{a}}_\mathbf{2}^{\mathbf{*}}={\mathbf{a}}_\mathbf{2}-0.075{\mathbf{a}}_\mathbf{1}$, respectively. Due to the higher genetic correlation between the intercept and the slope in RNM-HOM, the GEBV and subsequent SNP effects for the slope were more strongly corrected than in RNM-HET, because they contained more scaling effects. Once back-solved, the scale-corrected SNP effects for the slope were far less correlated with the intercept for both RNM-HOM (0.11) and RNM-HET (− 0.06) (Fig. 3b and d). Therefore, the correction appeared to remove the effect of scale-type G × E interactions on the SNP effects for the slope.

A region that was significantly associated with the scale-corrected slope (described later) is highlighted in red in all the plots in Fig. 3. Before the correction, several SNPs in this region were associated with the intercept, especially in RNM-HOM. The correction uncovered this region in RNM-HOM by lifting away the associations of these SNPs. This also occurred in RNM-HET, but to a lesser extent.

**Fig. 4:**
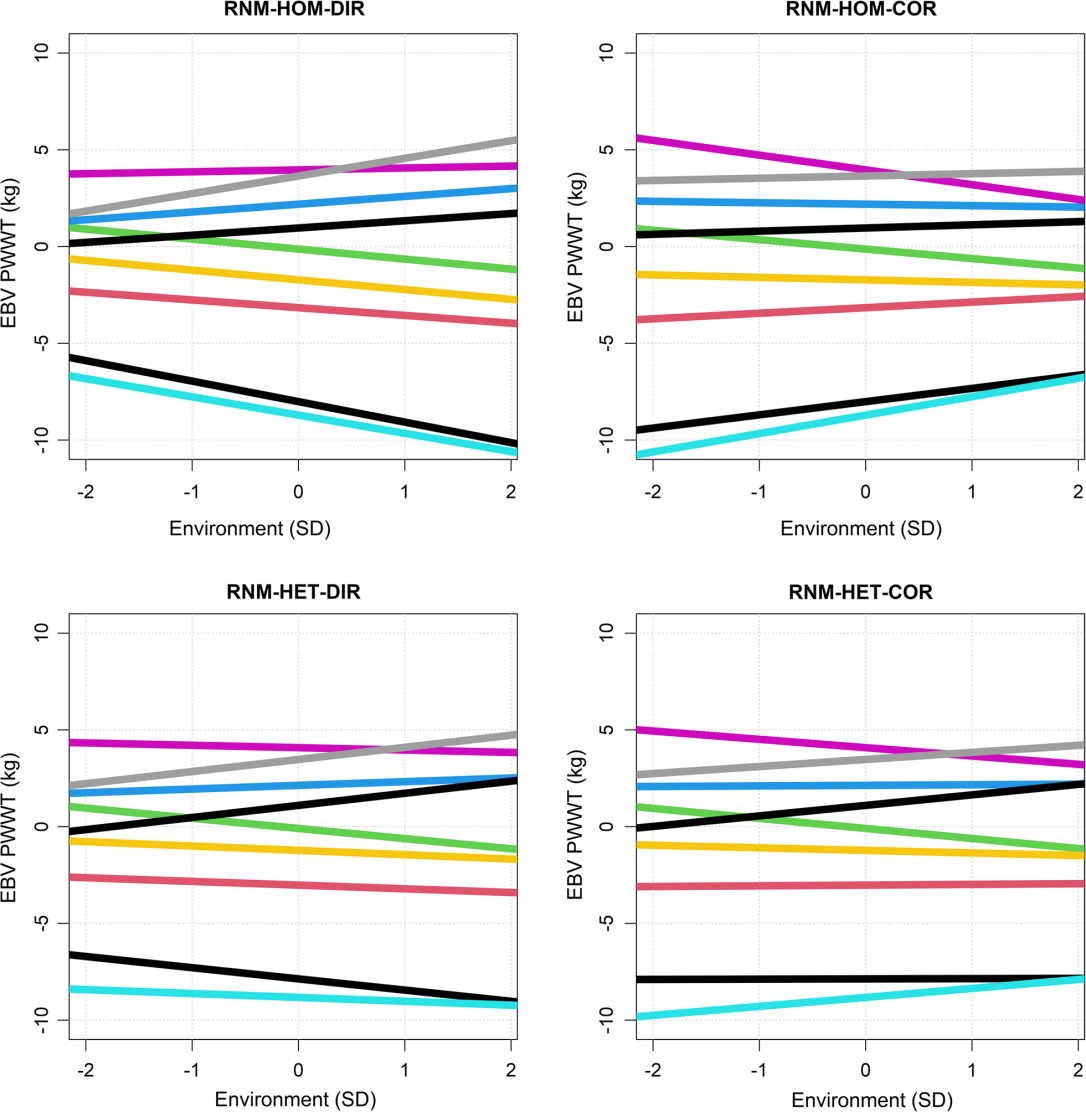
Reaction norms before and after scale-correction. Reaction norms for nine individuals representing the nine largest half-sib families with the largest distribution along the environmental gradient. Individuals are distinguished by colour. RNM-HOM-DIR: linear reaction norm model with homogenous residual and direct breeding value for slope; RNM-HOM-COR: linear reaction norm model with homogenous residual and corrected breeding value for slope RNM-HET-DIR: linear reaction norm model with heterogenous residual and direct breeding value for slope; RNM-HET-COR: linear reaction norm model with heterogenous residual and corrected breeding value for slope

The effect of the scale correction was also observed in the genomic reaction norms of genotypes before and after correction (Fig. 4). Genotypes with a large intercept effect had a greater correction in the slope. The sign of the slope also changed after correction for some genotypes.

**Fig. 5:**
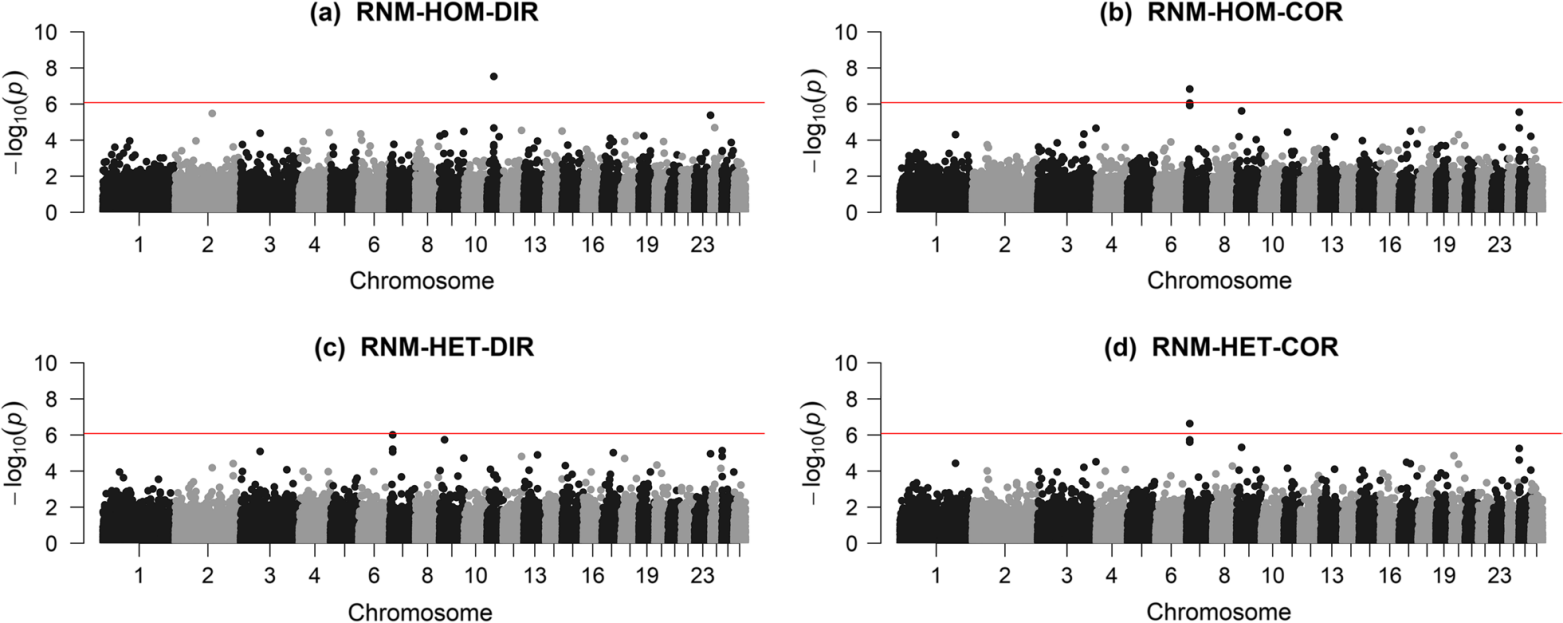
Genome-wide SNP associations for the direct and scale-corrected slope. Genome-wide SNP associations for the slope using direct (**a** and **c**) and scale-corrected (**b** and **d**) GEBV for the slope, for both RNM-HOM and RNM-HET. RNM-HOM-DIR: linear reaction norm model with homogenous residual and direct breeding value for slope; RNM-HOM-COR: linear reaction norm model with homogenous residual and corrected breeding value for slope; RNM-HET-DIR: linear reaction norm model with heterogenous residual and direct breeding value for slope; RNM-HET-COR: linear reaction norm model with heterogenous residual and corrected breeding value for slope

In RNM-HOM, Manhattan plots for the direct and scale-corrected slope detected different signals (Fig. 5a, b). The direct slope yielded a signal on chromosome 11, which was also associated with the intercept (see Additional file 7: Figure S3), but it disappeared when using the scale-corrected slope, and a new signal was detected on chromosome 7.

The same region on chromosome 7 was detected using the direct slope from RNM-HET, although it was not significant. However, this region was significant when using the scale-corrected slope in RNM-HET. In spite of significant differences between the Manhattan plots of the two models using the direct slope, the Manhattan plots for scale-corrected slope from both models were very similar, and the correlation between their SNP effects was very high [0.99, (see Additional file 8: Figure S4)]. Overall, the GWA analysis suggests that robustness is a highly polygenic trait that is regulated by many genes with small effects.

The three most significant SNPs associated with robustness were within an 881-bp region on chromosome 7 (Table 2). The allele substitution effect for these SNPs indicated that the animals with genotypes carrying the minor allele were approximately 4 g heavier at post-weaning for each one SD decrease in EG, compared to genotypes carrying the major allele. Only one of these SNPs was significant based on the Bonferroni-corrected p-value, which indicates a weak association. The three SNPs were located within the *SLC24A1* gene, which encodes a sodium/potassium/calcium exchanger. Eight other genes were located within a 500-kb region surrounding the most significant SNP (Table 3).

**Table 2:** SNPs associated with level of robustness

Chromosome	Base pair	MAF	− log_10_(p)	ASE (g/SD EG)
7	12,312,079	0.49	5.73	− 4.0
7	12,312,384	0.39	5.61	− 4.0
7	12,312,960	0.49	6.63	− 4.4

*MAF* minor allele frequency; *ASE* allele substitution effect, expressed in g per unit SD of the EG

p: Bonferroni-corrected p-value

**Table 3:** List of known genes in a 500-kb window surrounding the most significant SNP

Gene symbol	NCBI gene ID	Distance from the most significant SNP (kb)	Gene description
*SLC24A1*	101121857	0	Sodium/potassium/calcium exchanger
*INTS14*	101121097	30.0	Integrator complex subunit 14
*HACD3*	101120835	59.5	3-hydroxyacyl-CoA dehydratase 3
*DPP8*	101119728	106.5	Dipeptidyl peptidase 8
*IGDCC4*	101111715	189.1	Immunoglobulin superfamily DCC subclass member 4
*IGDCC3*	101111458	222.3	Immunoglobulin superfamily DCC subclass member 3
*DENND4A*	101122440	138.6	C-myc promoter-binding protein
*RAB11A*	780464	212.1	ras-related protein Rab-11A
*MEGF11*	101123125	240.6	Multiple epidermal growth factor-like domains protein 11

## Discussion

### Reaction norm models

Additive genetic variation was observed for the slope of the RNM, which indicated the presence of G × E interactions for PWWT in different growth environments. Both scale- and rank-type G × E interactions appeared to contribute to the variation in the slope, as evidenced by the differences in genetic variance along the EG and the much lower than 1 genetic correlations between environments. Other studies have identified significant G × E interactions for body weight of sheep using RNM [5, 26, 27], although the genetic correlations between environments found in our study are slightly lower than those in previous studies The estimates of heritability for PWWT in the best model, i.e., RNM-HET, were comparable to those obtained in previous studies for body weight in Australian sheep (0.31–0.39) [28, 29]. Overall, our findings demonstrate variation in the performance of sheep between growth environments, which can be captured using RNM.

We found that fitting heterogenous residual variance significantly improved the fit of the RNM, as reported in similar RNM studies of livestock [4, 27, 30]. When homogenous residual variance was assumed, the genetic variance increased by approximately 177% along the EG, which greatly over-estimated the amount of scale-type G × E interaction. Scale-type G × E interaction was detected at a much lower level after fitting heterogenous residual variance. This agrees with [31], who found that not accounting for existing heterogenous residual variance inflated the scale-type G × E interactions. In spite of the difference in scale-type G × E interactions, estimates of genetic correlation between environments were similar for the two models, RNM-HOM and RNM-HET. The low genetic correlation (0.44–0.49) suggested a large amount of re-ranking between environments, which was evident in Fig. 4, when the reaction norms of genotypes crossed over along the EG.

The genetic correlation between the intercept and the slope is commonly interpreted as a genetic correlation between overall performance and robustness, i.e., a high correlation indicates that genotypes that perform well tend to be more sensitive [32, 33]. This interpretation is problematic, as low-performing genotypes with negative intercepts will also have proportionally larger slopes (i.e., are equally as sensitive). Alternatively, a genetic correlation between the intercept and the slope could simply represent a scale effect, where some of the variance in GEBV along the EG is due to heterogenous genetic variance. This variation would not involve re-ranking and therefore might not reflect robustness. Thus, we used genetic regression to account for scale, where the GEBV for the slope and subsequent SNP effects were adjusted so that they were independent of the genetic correlation with the intercept. The same approach has been used in the analysis of residual feed intake [34].

### Genome-wide association analysis

The direct GEBV for the slope have been used in GWA studies to identify SNPs associated with robustness [6, 35, 36]. However, this approach could result in SNPs that are associated with the intercept masking the SNPs associated with re-ranking. This was demonstrated in RNM-HOM, for which the high correlation between SNP effects for the intercept and slope (0.85) masked the SNPs associated with reranking (Fig. 3a) and identified a region on chromosome 11 that affects robustness. Adjusting the GEBV to account for the scale-type G × E interactions by applying genetic regression reduced the correlation between the SNP effects considerably (0.11) and removed the signal on chromosome 11. The GWA analysis was able to reveal SNPs on chromosome 7 that were associated with robustness, independent of the scale effects. This phenomenon was also observed in RNM-HET, although it was less affected by scale. In this case, the region on chromosome 7 was non-significant when using the direct GEBV for the slope (− log_10_[p] = 5.98), but was significant after scale-correction (− log_10_[p] = 6.63). These findings suggest that correcting for scale effects on the GEBV for the slope can greatly influence the genomic regions detected and the interpretation of GWA analyses for robustness.

Interestingly, the correlation between the SNP effects for the intercept and scale-corrected slope in both models was not exactly zero. However, the genetic regression used for the scale-corrected slope was based on the estimated genetic (co)variance components, rather than the (co)variance of the GEBV used to back-solve the SNP effects. These (co)variances are not identical, as the genetic (co)variance components estimate the variance of the true breeding values (${\mathrm{V}}_{\mathrm{a}}$), while the variance of the GEBV is equal to ${\mathrm{V}}_{\mathrm{a}}\times {r}^{2}$, where $r$ is the accuracy of the GEBV. Unless the accuracies of the GEBV are all perfect (i.e., 1), the scale-correction might not result in a correlation of 0 between the back-solved SNP effects for the intercept and the scale-corrected slope. This could explain the small deviations from 0 found in this study.

Intuitively, the correlation between SNP effects estimated from the GEBV for the intercept and the slope are expected to be similar to the genetic correlation estimated in the RNM. However, the correlation between SNP effects for the intercept and the slope was considerably higher in both models, which may be explained by the fact that random regression models do not estimate GEBV for the intercept and the slope independently. Similar to a multi-trait model, they allow information from one trait (the intercept) to inform the GEBV of the other trait (the slope) through the covariance. This sharing of information can result in GEBV for the two traits having a higher correlation than the estimated genetic correlation [37]. It is conceivable that the RNM ‘borrowed’ a large amount of information from the intercept to estimate the GEBV for the slope, especially considering that there was more variation in the intercept than in the slope. This could explain the higher correlation of SNP effects that were back-solved from the GEBV compared to the genetic correlation estimated in the RNM. Unlike a multi-trait model, in which the two traits can be analysed independently, the intercept and slope of the reaction norm must be estimated simultaneously. Therefore, using the genetic regression which accounts for this correlation could be especially important when using random regression models to study the genomics of reaction norms.

One genomic region on chromosome 7 was significantly associated with the robustness of PWWT to different growth environments. Several genes identified in this region are associated with body weight and composition-related traits in livestock. The *SLC24A1*, *DENND4A* and *RAB11A* genes are associated with both average daily gain in Nellore cattle [38] and back-fat thickness in a composite cattle breed [39]. Similarly, *MEGF11* is associated with feed efficiency in cattle and pigs [40, 41], and body length in cattle [40]. Intriguingly, some of these genes have been implicated in disease-related traits. For example, Wang et al. [42] showed that *SLC24A1* is involved in respiratory and metabolic alkalosis in chickens under heat stress, and speculated that it may have a driving role in stabilizing the acid–base balance in the blood. *SLC24A1* has also been shown to be downregulated in cows with subclinical mastitis [43], while *MEGF11* is associated with somatic cell count in cattle [44]. In addition, the *IGDCC4* and *IGDCC3* genes encode antibodies [25], which are a critical component of the immune system. All of these pathways could be important when animals are raised in challenging environments.

The identification of these genes improves our understanding of the biology of robustness. However, the value of this information for increasing the accuracy of prediction of robustness is likely negligible, since the effect of these genes appears to be very small. Implementing selection for robustness based on GBLUP should efficiently capture these polygenic effects without the need to understand the underlying biology [45].

While the results of this study demonstrate the importance of considering the impact of scale-type G × E interactions when analysing robustness, the impact of the scale-correction in affecting selection decisions and genetic progress in breeding programs is less clear. A genotype which ranks highly across environments for traits such as body weight is evidently more valuable than a genotype which only ranks highly in specific environments. Simulations that explore selection for robustness under various scenarios of G × E interactions are needed to further understand the impact of the scale-correction, as well as the value of selecting for robustness in breeding programs.

## Conclusions

This study demonstrates the existence of important G × E interactions for PWWT of sheep in different growth environments and highlights the possibility of selecting sheep based on their robustness. Correction of slope breeding values using a genetic regression is suggested to account for scale-type G × E interactions on estimates of robustness. The genetic architecture of robustness in sheep appears to be highly polygenic.

## Supplementary Information

**Additional file 1: Table S1.** Breed composition of the dataset used to estimate contemporary group effects and reaction norm models.

**Additional file 2: Table S2.** Descriptive statistics for post-weaning growth rate (PWGR) and post-weaning weight (PWWT).

**Additional file 3: Figure S1.** Distribution of animals and mean post-weaning weight across the environmental gradient.

**Additional file 4: Table S3.** Approximate number of reference animals used for imputation for each SNP panel.

**Additional file 5: Figure S2.** Residual variance along the environmental gradient for the RNM-HET.

**Additional file 6: Table S4.** Comparisons of additive genetic variance (Table S4a), heritability (Table S4b) and genetic correlations (Table S4c) between the three growth environments (low, average and high).

**Additional file 7: Figure S3.** Manhattan plots for the intercept in the RNM-HOM and RNM-HET.

**Additional file 8: Figure S4.** Correlation between SNP effects for scale-corrected slope from the RNM-HOM and RNM-HET.

## Data Availability

The data are owned by Meat and Livestock Australia and access to the data can be negotiated by request.
