# Supplementary material for: Genomic analysis of the slope of the reaction norm for body weight in Australian sheep
Source: Genet Sel Evol. 2022 Jun 3;54:40. doi: 10.1186/s12711-022-00734-6 (PMC9164502; doi:10.1186/s12711-022-00734-6)
Supplement: Supplementary file 1 — Additional file 1: Table S1. Breed composition of the dataset used to estimate contemporary group effects and reaction norm models. [file 12711_2022_734_MOESM1_ESM.docx]

**Table S1 Breed composition of the INF and RF data used to estimate the contemporary group effects (a) and the reaction norm models (b)**

| **Breed** | **a** | **b** |
| --- | --- | --- |
| Merino | 60.8 | 60.3 |
| Border Leicester | 9.1 | 9.7 |
| Poll Dorset | 10.6 | 11.8 |
| Suffolk | 3.3 | 3.2 |
| White Suffolk | 3.6 | 4.1 |
| Research | 4.1 | 2.5 |
| Other | 8.5 | 8.4 |
