# Supplementary material for: Genomic analysis of the slope of the reaction norm for body weight in Australian sheep
Source: Genet Sel Evol. 2022 Jun 3;54:40. doi: 10.1186/s12711-022-00734-6 (PMC9164502; doi:10.1186/s12711-022-00734-6)
Supplement: Supplementary file 2 — Additional file 2: Table S2. Descriptive statistics for post-weaning growth rate (PWGR) and post-weaning weight (PWWT). [file 12711_2022_734_MOESM2_ESM.docx]

**Table S2 Descriptive statistics for post-weaning growth rate (PWGR) used to estimate contemporary group effects and the post-weaning weight (PWWT) before and after removing non-genotyped animals that were eliminated the reaction norm analysis.**

| **Trait** | **Male** | **Female** | **Mean** | **SD** | **Min** | **Max** |
| --- | --- | --- | --- | --- | --- | --- |
| PWGR (g/day) | 16 898 | 16 875 | 117.7 | 60.3 | -129.0 | 365.71 |
| PWWT (kg)^a^ | 16 898 | 16 875 | 43.4 | 9.4 | 9.0 | 79.5 |
| PWWT (kg)^b^ | 10 585 | 11 928 | 44.2 | 8.7 | 13.2 | 78.8 |

**^a^** Summary using all animals in the data. ^b^ Summary using genotyped animals used for the reaction norm analysis.
