# Supplementary figures and images for: Genomic analysis of the slope of the reaction norm for body weight in Australian sheep
Source: Genet Sel Evol. 2022 Jun 3;54:40. doi: 10.1186/s12711-022-00734-6 (PMC9164502; doi:10.1186/s12711-022-00734-6)

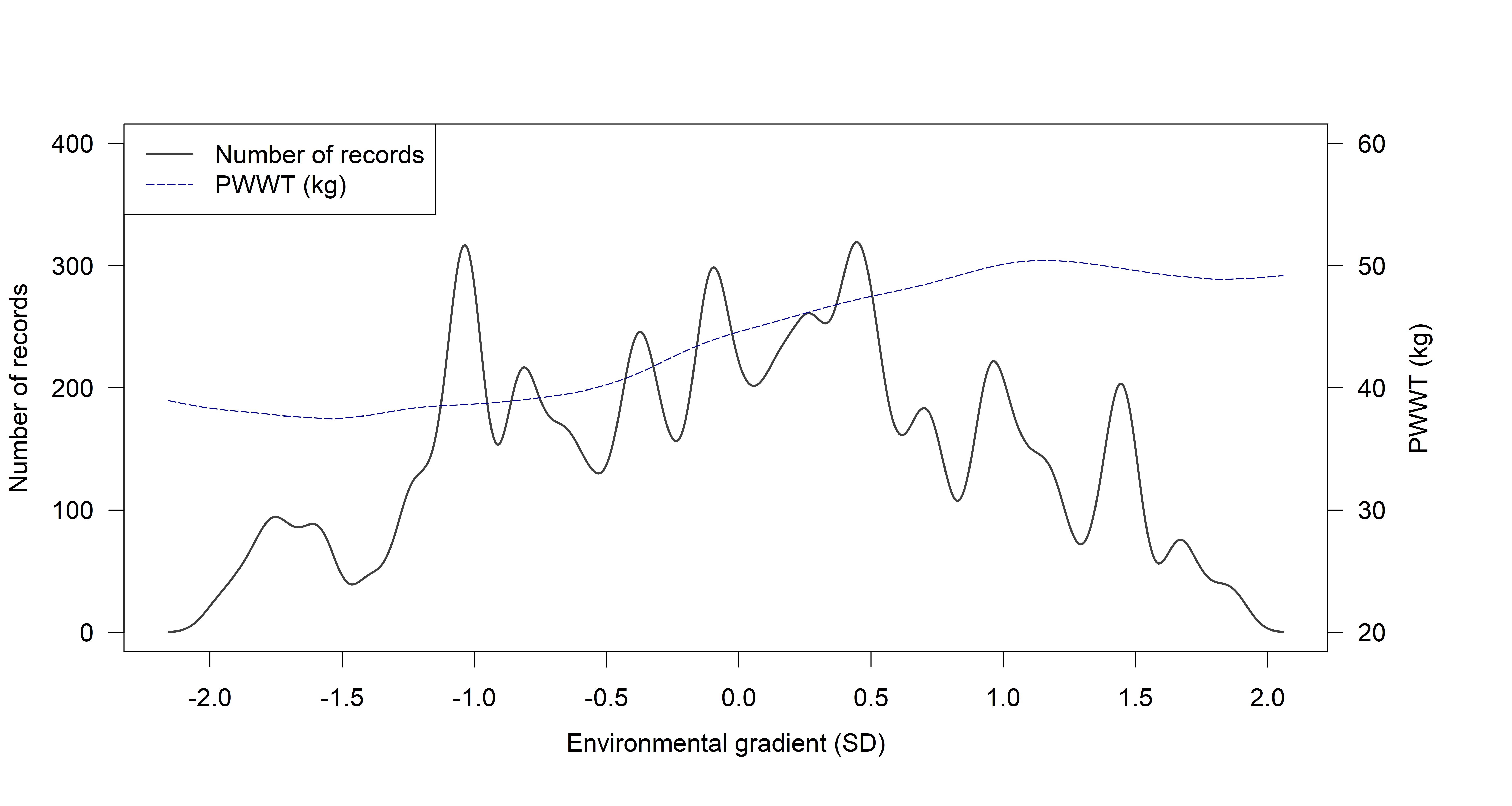

Supplement: Supplementary file 3 — Additional file 3: Figure S1. Distribution of animals and mean post-weaning weight across the environmental gradient. [file 12711_2022_734_MOESM3_ESM.png]

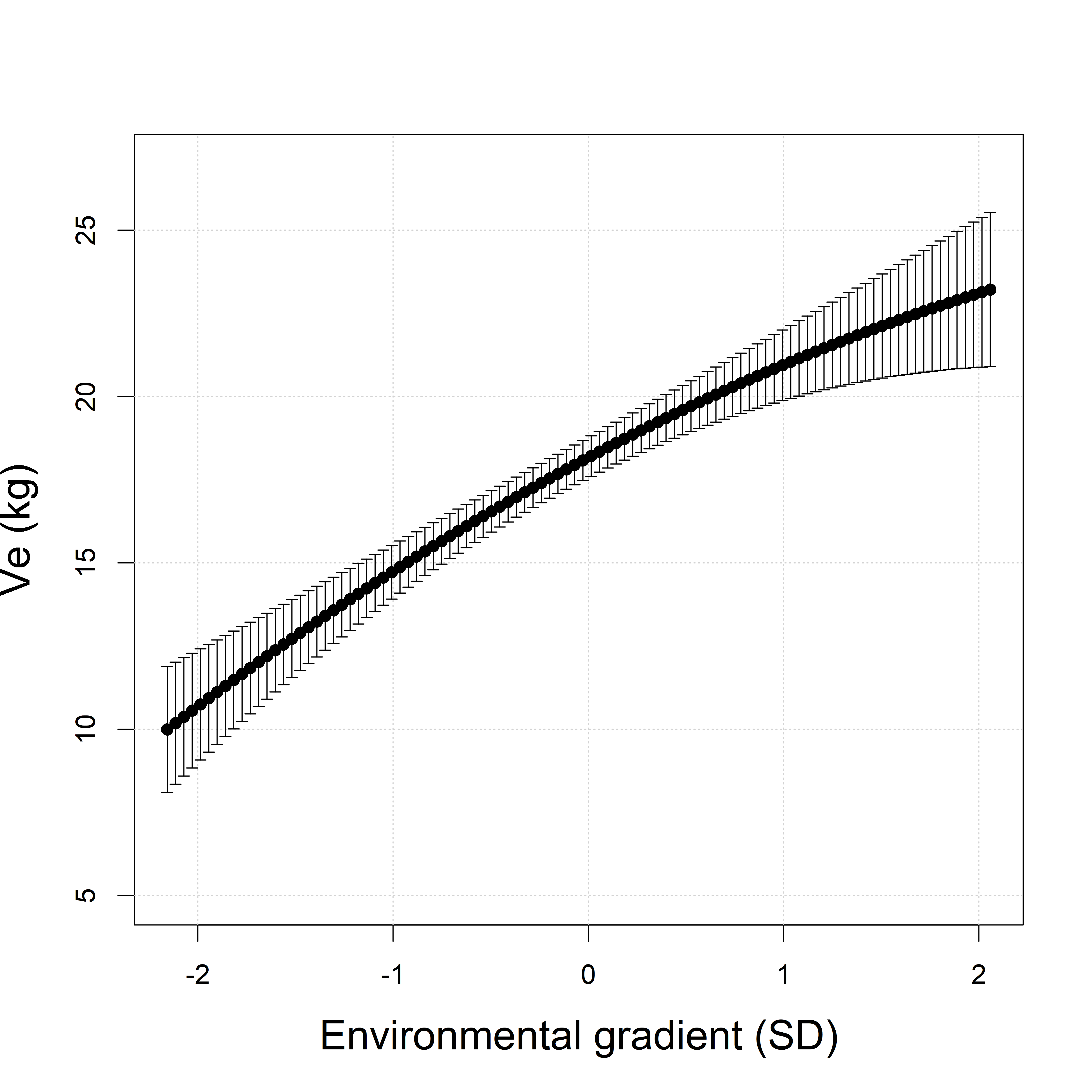

Supplement: Supplementary file 5 — Additional file 5: Figure S2. Residual variance along the environmental gradient for the RNM-HET. [file 12711_2022_734_MOESM5_ESM.png]

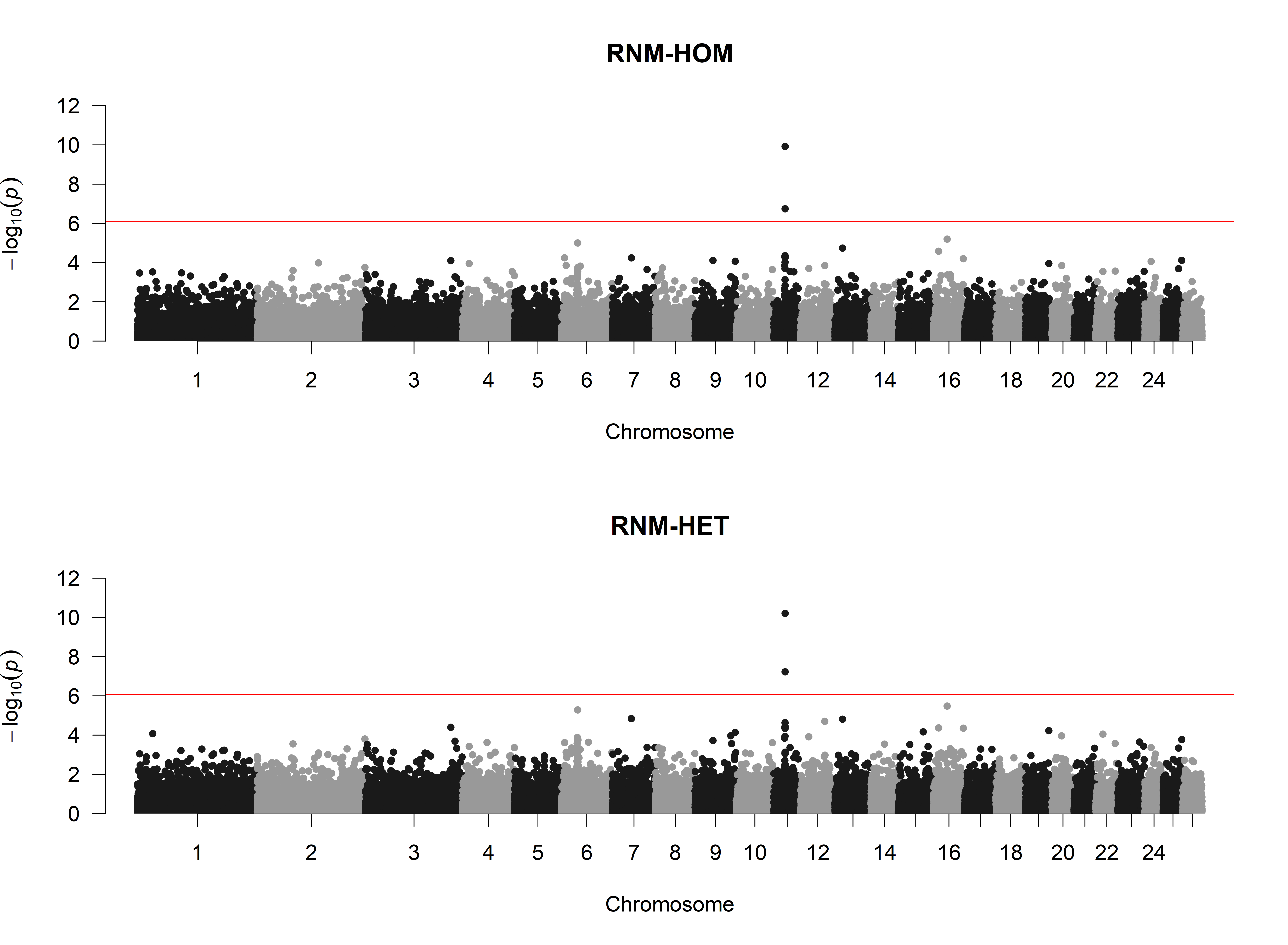

Supplement: Supplementary file 7 — Additional file 7: Figure S3. Manhattan plots for the intercept in the RNM-HOM and RNM-HET. [file 12711_2022_734_MOESM7_ESM.png]

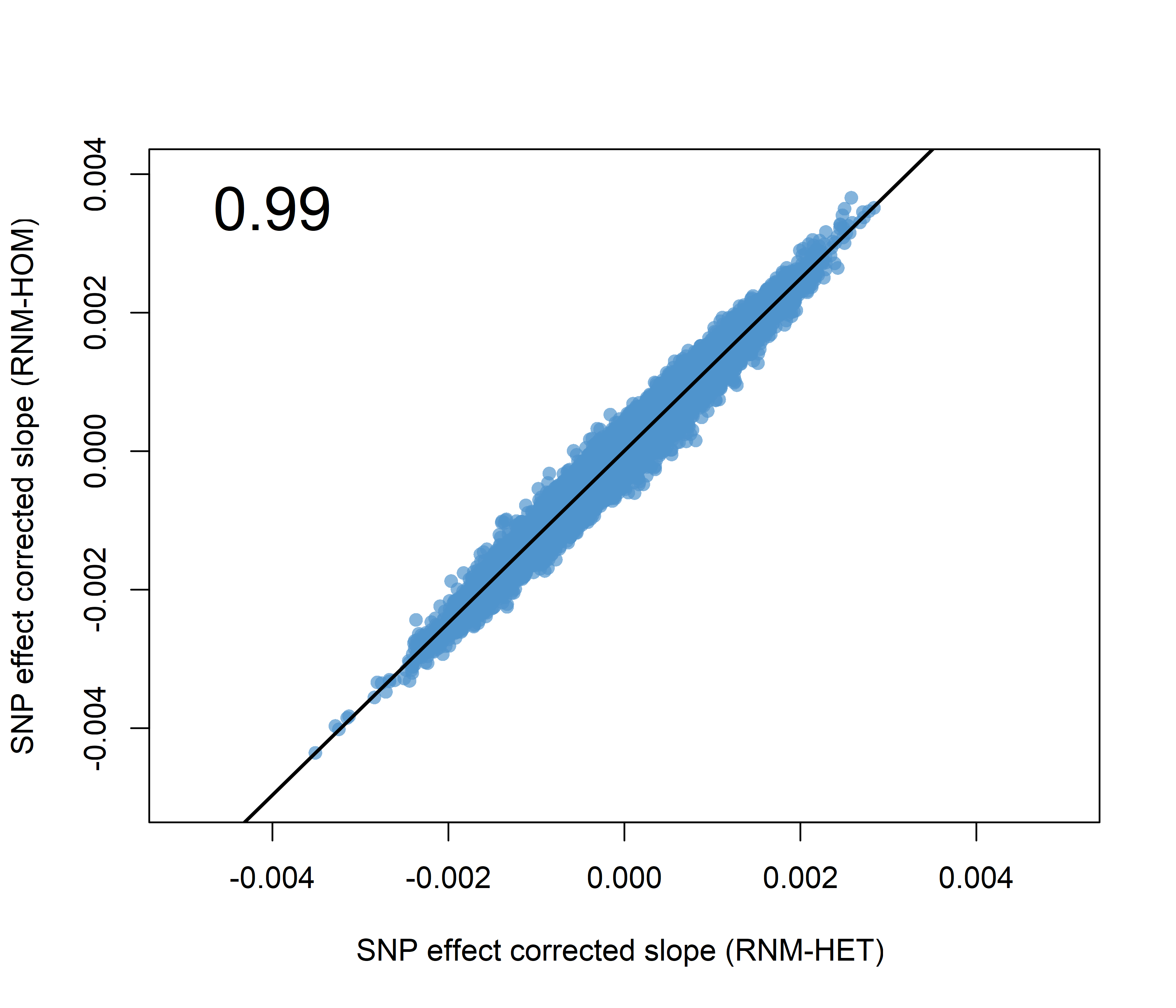

Supplement: Supplementary file 8 — Additional file 8: Figure S4. Correlation between SNP effects for scale-corrected slope from the RNM-HOM and RNM-HET. [file 12711_2022_734_MOESM8_ESM.png]
