# Supplementary material for: Genomic analysis of the slope of the reaction norm for body weight in Australian sheep
Source: Genet Sel Evol. 2022 Jun 3;54:40. doi: 10.1186/s12711-022-00734-6 (PMC9164502; doi:10.1186/s12711-022-00734-6)
Supplement: Supplementary file 4 — Additional file 4: Table S3. Approximate number of reference animals used for imputation for each SNP panel. [file 12711_2022_734_MOESM4_ESM.docx]

**Table S3 Approximate number of reference animals used for imputation for each SNP panel**

| **SNP panel** | **Number of reference animals** |
| --- | --- |
| 50k 1 | 183 000 |
| 50k 2 | 88 000 |
| 15k 1 | 62 000 |
| 15k 2 | 15 000 |
| 12k | 14 000 |
