# Supplementary material for: Genomic analysis of the slope of the reaction norm for body weight in Australian sheep
Source: Genet Sel Evol. 2022 Jun 3;54:40. doi: 10.1186/s12711-022-00734-6 (PMC9164502; doi:10.1186/s12711-022-00734-6)
Supplement: Supplementary file 6 — Additional file 6: Table S4. Comparisons of additive genetic variance (Table S4a), heritability (Table S4b) and genetic correlations (Table S4c) between the three growth environments (low, average and high). [file 12711_2022_734_MOESM6_ESM.docx]

**Table S4a Additive genetic variance for each environment (low, average and high growth environments) in the three different models**

| **Environment** | **Model^a^** | | |
| --- | --- | --- | --- |
|  | **RNM_HOM** | **RNM_HET** | **MT** |
| Low | 5.46 (0.45) | 8.12 (0.60) | 9.31 (0.97) |
| Average | 7.45 (0.32) | 7.00 (0.30) | 7.04 (0.49) |
| High | 15.14 (0.76) | 11.25 (0.80) | 13.57 (1.35) |

^a^ RNM-HOM: linear reaction norm with homogenous residual variance; RNM-HET: linear reaction norm with heterogenous residual variance; MTM: Multi-trait model which considers performance in the three environments as separate but correlated traits with heterogenous residual variance.

**Table S4b Heritability for each growth environment (low, average and high) in the three different models**

| **Environment** | **Model^a^** | | |
| --- | --- | --- | --- |
|  | **RNM_HOM** | **RNM_HET** | **MT** |
| Low | 0.24 (0.02) | 0.39 (0.03) | 0.31 (0.02) |
| Average | 0.30 (0.01) | 0.28 (0.01) | 0.23 (0.01) |
| High | 0.46 (0.01) | 0.34 (0.02) | 0.29 (0.02) |

^a^ RNM-HOM: linear reaction norm with homogenous residual variance; RNM-HET: linear reaction norm with heterogenous residual variance; MTM: Multi-trait model which considers performance in the three environments as separate but correlated traits with heterogenous residual variance.

**Table S4c Genetic correlations between each growth environment (low, average and high) in the three different models**

| **Environment** | **Model^a^** | | |
| --- | --- | --- | --- |
|  | **RNM_HOM** | **RNM_HET** | **MT** |
| Low vs average | 0.77 | 0.81 | 0.62 |
| Average vs high | 0.92 | 0.88 | 0.81 |
| Low vs high | 0.49 | 0.44 | 0.67 |

^a^ RNM-HOM: linear reaction norm with homogenous residual variance; RNM-HET: linear reaction norm with heterogenous residual variance; MTM: Multi-trait model which considers performance in the three environments as separate but correlated traits with heterogenous residual variance.
